# Phytohormones Producing *Acinetobacter bouvetii* P1 Mitigates Chromate Stress in Sunflower by Provoking Host Antioxidant Response

**DOI:** 10.3390/antiox10121868

**Published:** 2021-11-24

**Authors:** Muhammad Qadir, Anwar Hussain, Muhammad Hamayun, Mohib Shah, Amjad Iqbal, Muhammad Irshad, Ayaz Ahmad, Muhammad Arif Lodhi, In-Jung Lee

**Affiliations:** 1 Department of Botany, Garden Campus, Abdul Wali Khan University Mardan, Khyber Pakhtunkhwa, Mardan 23200, Pakistan; muhammad_qadir@awkum.edu.pk (M.Q.); hamayun@awkum.edu.pk (M.H.); mohibshah@awkum.edu.pk (M.S.); muhammad.irshad@awkum.edu.pk (M.I.); 2 Department of Food Science & Technology, Garden Campus, Abdul Wali Khan University Mardan, Khyber Pakhtunkhwa, Mardan 23200, Pakistan; amjadiqbal@awkum.edu.pk; 3 Department of Biotechnology, Garden Campus, Abdul Wali Khan University Mardan, Khyber Pakhtunkhwa, Mardan 23200, Pakistan; ahdayazb5@awkum.edu.pk (A.A.); arif.lodhi@awkum.edu.pk (M.A.L.); 4 Department of Applied Biosciences, Kyungpook National University, Daegu 41566, Korea

**Keywords:** Cr^+6^ bioremediation, improved antioxidant activity, plant growth promotion, rhizobacteria

## Abstract

Different physical and chemical techniques are used for the decontamination of Cr^+6^ contaminated sites. The techniques are expensive, laborious, and time-consuming. However, remediation of Cr^+6^ by microbes is viable, efficient, and cost-effective. In this context, plant growth-promoting rhizobacteria *Acinetobacter bouvetii* P1 isolated from the industrial zone was tested for its role in relieving Cr^+6^ induced oxidative stress in sunflower. At the elevated Cr^+6^ levels and in the absence of P1, the growth of the sunflower plants was inhibited. In contrast, the selected strain P1 restored the sunflower growth under Cr^+6^ through plant growth–promoting interactions. Specifically, P1 biotransformed the Cr^+6^ into a stable and less toxic Cr^+3^ form, thus avoiding the possibility of phytotoxicity. On the one hand, the P1 strengthened the host antioxidant system by triggering higher production of enzymatic antioxidants, including catalases, ascorbate peroxidase, superoxide dismutase, and peroxidase. Similarly, P1 also promoted higher production of nonenzymatic antioxidants, such as flavonoids, phenolics, proline, and glutathione. Apart from the bioremediation, P1 solubilized phosphate and produced indole acetic acid, gibberellic acid, and salicylic acid. The production of phytohormones not only helped the host plant growth but also mitigated the harsh condition posed by the elevated levels of Cr^+6^. The findings mentioned above suggest that P1 may serve as an excellent phyto-stimulant and bio-remediator in a heavy metal-contaminated environment.

## 1. Introduction

Heavy metals (HMs) are natural components of the earth’s crust. However, their biogeochemical cycles and biochemical balances are disrupted due to rapid industrialization. Such immense industrial activities led to a higher increase and subsequent aggregation of HMs in the milieu. Additionally, the elevated levels of HMs in water, soil, and agricultural products are the result of excessive but imprudent agricultural practices, industrial and domestic wastes [1,2]. The accumulation of HMs in plants affects humans directly through the consumption of contaminated plant-based foods. Heavy metals contaminated plants can also affect humans indirectly through the consumption of meat and milk from livestock fed on such plants. Upon exposure, HMs affect cell organelles, including cell membrane, nucleus, endoplasmic reticulum, mitochondria, lysosome, enzymes, and metabolites involved in the detoxification mechanisms and injury recovery [3]. Interaction of HMs with vital cell components such as DNA and proteins leads to their damage, conformational changes, cell cycle modulation, oncogenesis, and programmed cell death [4,5]. Based on significant public health concerns, the remediation of heavy metals should take priority [6].

Amid the HMs, hexavalent chromium (Cr^+6^) is a widespread environmental pollutant mainly from chromium-dependent industries. In undeveloped countries, industrial wastewater is released into natural water resources, most of which are not adequately treated and pose significant threats to the ecosystem. Hexavalent chromium (Cr^+6^) is more toxic and oncogenic, and these properties are due to their higher solubility in water, high permeability through biofilms, and subsequent interaction with inter and intracellular proteins and nucleic acids [7].

In plants, metal-induced phytotoxicity and generation of reactive oxygen and nitrogen species have revealed that redox-active metals, particularly chromium (Cr), undertake redox cycling reactions. In fact, chromium owns the potential to produce reactive species, such as anions of superoxide and nitric oxide radicals in biological systems [8]. Moreover, the disturbed metals homeostasis may lead to oxidative pressure, a state where the imbalance production of reactive oxygen species (ROS) overwhelms the antioxidant defense [9]. Such conditions may cause DNA and chromosomal damage, malonaldehyde production, conformational changes in protein structure, and subsequent toxicity. The damages in the host plants due to oxidative injuries, enhanced lipid peroxidation, and altered calcium and sulfhydryl homeostasis can be translated into stunted growth. Lipid peroxides, formed by the attack of radicals on polyunsaturated fatty acid residues of phospholipids, can produce mutagenic and carcinogenic malondialdehyde, 4-hydroxynonenal, and other exocyclic DNA adducts (etheno and propano adducts) [9].

Anionic transporters in plants may take up Cr^+6^ in the form of chromate. Cr^+6^ is easily converted to Cr^+3^ under most environmental conditions. Cr in its +3 form was once thought to be a vital ingredient for animal and human existence. The role of Cr^+3^ in the glucose tolerance factor, as well as the beneficial effects of Cr^+3^ dietary supplements on diabetes and lipid metabolism, have been studied in the past [10]. Quiet recently, Cr^+3^ has been removed from the list of essential elements for humans and animals due to a lack of clear experimental evidence regarding its function in humans and animals [11].

In the current scenario, remediation of contaminated water bodies and soil is the call of the day to remove heavy metals and trace elements from the human environment in order to make it healthy. Chemical precipitation, oxidation or reduction filtration, ion exchange, reverse osmosis, membrane technology, evaporation, and electrochemical treatment are only a few methods for eliminating these heavy metals [12]. These methods, however, are usually useless when heavy metal concentrations are lower than 100 mg/L [13]. Because salts of heavy metals are readily soluble and dissolve in water, the physical separation techniques are not feasible to separate them. Furthermore, at minimal metal concentrations, physical and chemical techniques are inefficient or costly [13]. In contrast to physicochemical approaches, bioremediation (biosorption and bioaccumulation) may possibly present the optimal strategy for heavy metal elimination [14]. As a result, using microbes and plants to repair and rebuild the natural conditions of the soil is possible and sustainable. Microbial communities’ responses to heavy metals depend on their concentration and availability, which is a complicated process influenced by the type of the metal, medium characteristics, and microbial species [15].

Bioremediation is a cutting-edge and promising technique for removing and recovering heavy metals from contaminated water and agrarian soil [16]. Microbes develop and utilize several detoxification processes as a result of their varied survival approaches in heavy metal-contaminated environments [17]. Biosorption, bioaccumulation, biotransformation, and biomineralization are the primary detoxification processes that could be employed for bioremediation either in ex situ or in situ conditions [18]. Polysaccharides, lipids, and proteins make up the majority of microbial cell walls, and they include various side chains that chemically bind metal ions, including carboxylate, hydroxyl, and amino acids and phosphate groups [19].

Plant growth-promoting rhizobacteria (PGPR) is an environmentally benign and cost-effective bioremediation technology for reducing heavy metal pollution in soil [20]. Metal-tolerant PGPR also affects plant survival and adaptability through nitrogen fixation, phytohormone synthesis, and physicochemical changes in polluted soil [21,22]. Plant colonization by PGPR has been driven by the release of various compounds, amino acids, proteins, and antibiotics that aid plants in the removal of heavy metal toxicity. By reducing, oxidizing, methylating, or demethylating, compartmentalizing, and converting to a less hazardous form, the PGPR have the ability to reduce the harmful influence of heavy metals [23,24]. Cr^+6^ undergoes a transition to Cr^+3^, which is more stable, less water-soluble, and hence less harmful. Consequently, converting Cr^+6^ to Cr^+3^ is a promising approach for removing Cr^+6^ from the environment [25]. As a result, decontaminating Cr^+6^ using naturally existing microorganisms is a viable alternative. Cr^+6^-induced phytotoxicity, on the other hand, may be reduced by using plant growth-promoting bacteria. 

Furthermore, naturally existing rhizosphere microorganisms in soils contaminated with Cr^+6^ have developed resistance mechanisms to these metals’ toxicity [26,27]. The Cr^+6^-tolerant bacteria possess the substantial capacity to produce plant growth regulators, to solubilize inorganic P (salt of phosphoric acid with metals) fixation of free atmospheric N, and synthesize antimicrobial compounds and siderophores [28]. Recent studies have shown that chromate-tolerant rhizobacteria have the ability to cooperate with the host plant in many ways. Indeed, the Cr^+6^ tolerant rhizobacteria improve the host endogenous pool of phytohormones, strengthen the enzymatic and non-enzymatic arms of the antioxidant system, cut down metal uptake, and reduce the significant proportion of the accumulated metal to nontoxic or the least toxic form [29,30,31]. 

The area of study on the bioremediation of chromium from the agricultural soils by PGPR is still expanding. The present research aims to contribute to this growing body of knowledge by focusing on the premise that rhizobacteria can mitigate heavy metal stressful condition in the host plants. The objectives of the study were to establish (a) the function of PGPR in mitigation of Cr^+6^ induced oxidative stress in sunflower; (b) the effect of rhizobacterial inoculum on Cr^+6^ accumulation and translocation in the host; and (c) to explore the detoxification impact of PGPR on *Helianthus annuus* L. physiology and development under Cr^+6^.

## 2. Materials and Methods

### 2.1. Test Plant Species (Biological Materials)

Sunflower (*H. annuus* L.), native to America and commercialized in Russia as a crop plant, is a member of the Asteraceae family. It is a significant commercial crop that ranks fourth in the world among vegetable oil seeds after soybean, oil palm, and canola [32]. In Mardan, Pakistan, HySun-33 is one of the most widely cultivated varieties of sunflower. Seeds of sunflower HySun-33 were obtained from local agriculture research Centre.

### 2.2. Requisition of Rhizbacterium

A Cr^+6^ resistant strain of rhizobacterium P1 (Accession No# MT478039) was acquired from the bacterial stock from the Lab of Plant Microbes Interaction, Department of Botany, Abdul Wali Khan University Mardan. The rhizobacteria were previously isolated from the rhizosphere of *Parthenium hysterophorus* L. grown on metal-polluted soil in the locality of Premier Sugar Mill Mardan [33].

### 2.3. Molecular Identification of Strain

The 16S rDNA marker was used for the molecular identification of the isolate. Using a Gen-Elute DNA kit (Sigma-Aldrich, Burlington, MA, USA), genomic DNA was extracted from a selected strain culture grown overnight. The GeneAmp 9700 PCR system (Thermo Fisher Scientific) was used to conduct amplification using a set of universal primers FDD2 (5’-CCG GAT CCG TCC ACA GAG TTT GAT CTT GGC GAA -3’) and RPP2 (5’-CCA AGC TTC TAG ACG GAT ACC TTG TTA CGA CTT- 3’) (Applied Biosystems Inc., Atlanta, GA, USA). The amplified fragment was subjected to sequencing through the DNA sequencing service of MACROGEN, Korea (http://dna.macrogen.com/eng accessed on 6 May 2019) [34]. Reads obtained were used to generate consensus sequence through Codon Code Aligner (version 7.2.1, Codon Technology Corporations, Hyderabad, India). The consensus sequence was used as BLAST query to find closely matching sequences in NCBI’s GenBank database (https://blast.ncbi.nlm.nih.gov/Blast.cgi?PAGE_TYPE=BlastSearch accessed on 20 May 2019). Closely resembling sequences retrieved from the database were used to align with the selected sequence, and a phylogenetic tree was constructed using MEGA 7 (version 7.0.18, Pennsylvania State University, Pennsylvania, PA, USA).

### 2.4. Screening the Rhizobacterium

The isolate was tested for chromate tolerance, ability to release phosphate solubilizing substances, and for chromate stress alleviation in sunflower.

#### 2.4.1. Chromate Tolerance

To check the capability of the strain to endure elevated Cr^+6^ levels, the selected rhizobacterium was subjected to 100, 300, 500, 900, and 1200 µg mL^−1^ Cr^+6^ (in the form of K_2_CrO_4_ (Sigma Aldrich)) in Luria-Bertani (L.B) broth medium (Sigma Aldrich), while the nonmetal media were used as control. The cultures were kept in a shaker (Modle-Wis 20) for incubation at 130 rpm for 24 h at 28 °C. Bacterial growth in the control and metal supplemented media was recorded at 595 nm by monitoring the culture’s optical density (OD).

#### 2.4.2. Phosphate Solubilization Index

To assess the phosphate solubilization activity, the strain was grown on Pikovskaya’s agar medium (HiMedia™ Laboratories). In the aseptic environment, a spot inoculation of the bacterial isolate was made on the plates and incubated at 28 °C for 7 days. Uninoculated plate with PKV agar acted as a control. Comparative calculation of the solubilization index was conducted on day 7 of incubation by calculating transparent zone and colony diameters in centimeters. The activity of the phosphate solubilization index was calculated by the method of [35] using the following formula:

(1) $$\mathrm{Solubilization}\;\mathrm{index}=\frac{\mathrm{Colony}\;\mathrm{diameter}+\mathrm{Halozone}\;\mathrm{diameter}}{\mathrm{Colony}\;\mathrm{diameter}}\;$$

#### 2.4.3. Potential of the Isolate to Mitigate Chromate stress in Sunflower

*Helianthus annuus* seeds of uniform vigor and health were surface sterilized with 0.1% HgCl_2_ (Medical Supplier Uk) for 5 min and rinsed thrice with sterile dH_2_O to remove traces of HgCl_2_. The sterilized seeds were then placed on wet filter paper and supplemented with the following treatments in Petri plates:

Treatment 1 = 0 µg mL^−1^ of Cr^+6^
Treatment 2 = 100 µg mL^−1^ of Cr^+6^
Treatment 3 = 300 µg mL^−1^ of Cr^+6^
Treatment 4 = Bacterial inoculum.
Treatment 5 = Bacterial inoculum + 100 µg mL^−1^ of Cr^+6^
Treatment 6 = Bacterial inoculum + 300 µg mL^−1^ of Cr^+6^.

Each plate contained 10 seeds. Bacterial inoculum was prepared by harvesting overnight broth culture through centrifugation at 896 rcf for 20 min and resuspending the washed cells in 10 mM MgCl_2_. Inoculum density was adjusted to 10^−6^ cells/mL. Each treatment was applied to a set of three Petri plates.

### 2.5. Plant Growth Related Metabolites in Bacterial Culture

Rhizobacteria are known to produce a number of metabolites that help in their ability to interact with the host plant species to improve their growth. In this connection, plant hormones (indole 3-acetic acid, gibberellic acid, and salicylic acid), primary metabolites (proteins), and stress-related metabolites (phenolics, flavonoids, and proline) were assessed in bacterial culture. For this purpose, the rhizobacteria were cultured in L.B broth and incubated in a shaker at 30 °C and 130 rpm. Bacterial culture supernatant (BCS) was obtained by centrifuging (Sartorius Modle: 2–16 PK) the broth culture, which was then used to determine the metabolites mentioned above as discussed below:

#### 2.5.1. Determination of Phytohormones

Indole acetic acid was estimated in the BCS using the Salkowski reagent as described earlier [36]. Different known quantities of IAA (10–100 µg) (Sigma Aldrich, Burlington, MA, USA) were used to develop the calibration curve.

For the determination of GAs (gibberellins) in BCS, a wheat endosperm assay was performed. Wheat seeds of good health (10 embryo-less wheat seeds) were disinfected by treating them with 70% ethanol for 1 min, followed by washing with dH_2_O to remove ethanol traces. The seeds were placed in plates containing 2 mL of filter-sterilized BCS and acetate buffer (10 mL, pH 4.5). After incubation in the dark for 2 days at 28 °C, the buffer was recovered from Petri plates and tested for sugars by mixing with 200 µL of Benedict’s solution. The reaction mixtures were incubated for 30 min at room condition, and OD was recorded at 254 nm against a blank (all above material without BCS). The calibration curve was constructed by treating known concentrations of GA_3_ (Sigma Aldrich, Burlington, MA, USA) in the same manner mentioned and presented as GA_3_ equivalent [37].

To determine salicylic acid (SA) in BCS, the technique of Worrier et al. [38] was used. Different quantities (10–1000 µL) of the BCS were added to 1 mL of ethanol, and the mixture was centrifuged at 5600 rcf for 10 min. The supernatant was chilled at 4 °C in a refrigerator before estimating the SA. Approximately 100 µL of the supernatant was added to 2.9 mL of fresh FeCl_3_ (0.1%) solution (Sigma Aldrich, Burlington, MA, USA), and the absorbance was observed at 540 nm. Different quantities (10–100 µg) of SA (Sigma Aldrich, Burlington, MA, USA) were used to construct the standard curve.

#### 2.5.2. Flavonoids, Phenol, and Proline Determination

The aluminum chloride method was followed to estimate total flavonoid (TF) BCS [39]. Briefly, 500 μL of the supernatant was added with 100 μL of 10% AlCl_3_ (Aluminum chloride (Sigma Aldrich, Burlington, MA, USA), 100 μL of 10% potassium acetate, and 4.8 mL of 80% methanol. The blend was vortexed and kept in an incubator for 30 min incubation. After incubation, the optical density (OD) was noted at 415 nm. Various Quercetin (10–100 µg) concentrations (TargetMol, Boston, MA, USA) were used for plotting the calibration curve.

The phenols in the BCS were estimated by minor changes in the Folin–Ciocalteu method [40]. In short, to the 0.2 mL of bacterial culture supernatant, 0.8 mL of Folin–Ciocalteu reagent (MilliporeSigma, Bangalore India) and 2 mL of 7.5% Na_2_CO_3_ (sodium carbonate (Sigma Aldrich, Burlington, MA, USA)) was added. The sample was diluted to 7 volumes with dH_2_O and incubated for 2 h in the dark. Different concentration of catechol was used to develop a standard curve. The absorption was recorded at 765 nm against blank. Catechol (CellMark AB, Göteborg, Sweden) concentrations (1–10 mg) were used for developing a standard graph.

The technique of Bates et al. [41] was used to estimate proline concentration in the BCS. Approximately 100 µL from BCS was added to 3% sulfosalicylic acid (4 mL) (New Alliance Fine Chem Private Limited, Mumbai, India) followed by centrifugation at 504 rcf for 5 min. The supernatant was collected, added 2 mL of acidic ninhydrin, and incubated at 100 °C for 1 h. Once cooled, the reaction mixture and proline were separated through 4 mL of toluene in a separatory funnel. Optical density was documented at 520 nm using the blank (toluene). Different proline (SENOVA Technology Co., Ltd, Cambridge, UK) quantities (10–100 µg) were used for developing standard graphs.

### 2.6. Microbial Interaction with Host; a Strategy to Cop Excess Chromate

Healthy and good quality seeds of sunflower were surface disinfected, as mentioned earlier. Upon germination in sterilized sand, the sunflower seedlings were moved to pots with the Hoagland’s (400 mL) of half strength. The selected isolate P1 was subjected to test their capacity to relieve the chromate stress in plants. The pots were stored with 3 replicates (each having 4 seedlings) in a randomized complete block design (RCBD). Treatments had two main factorial variations: chromate levels (0, 25, 50, and 100 µg mL^−1^ of Cr^+6^) and inoculum of rhizobacterium. Plants were maintained under 28 °C, the humidity of 68%, the light intensity of 647.5 µmol m^−2^ s^−1^, and 13 h of photoperiod in LabTech (Model; LGC-5101 G) growth chamber. The seedlings were collected after 16 days at the 4–6 leaves stage. The relative growth rate (RGR) [42], net assimilation rate (NAR) [43], P1 colonization, and various growth attributes were recorded at the end of the experiment.

(2) $$\mathrm{RGR}=\frac{\mathrm{lnW}2\;-\;\mathrm{lnW}1\;}{t\;2\;-\;t\;1\;}$$

where ln is the natural log, W1 and W2 are plant dry weights at times t1 and t2.

(3) $$\mathrm{NAR}=\frac{W\;2\;-\;W\;1}{t\;2\;-\;t\;1\;}\;\times \;\frac{\mathrm{In}\;L\;2\;-\;\mathrm{In}\;L\;1\;}{L\;2\;-\;L\;1\;}$$

where W2 and W1 are the plant dry weights at times t1 and t2, logeA2 and logeA1 are the natural logs of leaf areas A1 and A2 at times t1 and t2.

The experiment was repeated three times, and the data were pooled and analyzed statistically.

### 2.7. Determination of Plant’s Metabolites

To understand plant physiological and biochemical responses under chromate stress, different metabolites were tested in the plants harvested in the experiment mentioned above. The following metabolites were estimated in seedling biomass and in exudation by their roots.

#### 2.7.1. Estimation of Indole Acetic Acid

The plant material (leaves) used for the analysis of phytohormones were ground in liquid nitrogen. For extraction of indole acetic acid, 3–10 g of the powder was homogenized (in case of exudates 3–10 mL) in 100% methanol (2 mL/g of plant sample) and centrifuged at 5600 rcf. The supernatant was used to determine indole acetic acid using the Salkowski reagent method as mentioned above [36].

#### 2.7.2. Estimation of Total Flavonoids, Total Phenols, and Proline

To extract total flavonoids, 0.5 g of leaves (0.5 mL in case of exudates) were crushed in 5 mL of 80% aqueous ethanol and incubated for 24 h in a shaking incubator. The extract was then centrifuged for 15 min at 5600 rcf and 25 °C. The supernatant containing flavonoids was used for the estimation of flavonoids by the AlCl_3_ technique as described above [39].

Total phenolics were extracted by homogenizing 1 g of leaves (or 1 mL exudates) in 16 mL of ethanol. The samples were incubated at 20 °C to 80 °C for 3 h. Following incubation, we centrifuged the samples for 10 min at 5600 rcf. The samples were filtered, concentrated at 40 °C to 1 mL, and re-dissolved in 10 mL distilled water and stored at 4 °C prior to use. Total phenolics were estimated by the technique mentioned previously [44].

For the extraction of proline, 200 mg of leaves were homogenized in 1 mL of ethanol/water (40:60 *v*/*v*) and incubated for 24 h at 4 °C. The incubation was followed by centrifugation at 5600 rcf (5 min). For 100% extraction of proline, the process was repeated and estimated as mentioned above [41].

#### 2.7.3. Malondialdehyde (MDA) Determination

Schmedes and Hølmer [45] method was followed to estimate MDA concentration in the host leaves. Approximately 0.2 g of leaves were crushed in 2 mL of 0.6% thiobarbituric acid (Sigma-Aldrich, Burlington, MA, USA). Centrifugation of the samples was done for 10 min at 8064 rcf followed by incubation at 100 °C for 15 min in a water bath. Following incubation, the samples were cooled down and centrifuged again at 8064 rcf. The optical density of the samples was measured at 532, 600, and 450 nm. The calibration curve was constructed in the concentration range of 0.1 to 1.0 mM of MDA (Sigma-Aldrich, Burlington, MA, USA).

### 2.8. Estimation of Electrolyte Leakage

To remove surface adhered electrolytes, the leaves from a 7-days stressed seedlings were washed thrice with deionized water. After rinsing, discs of equal size were scraped out of the leaves to determine electrolyte leakage. The scraped discs were then transferred to capped vails having 10 mL of dH_2_O and incubated at 120 rpm and 25 °C for 24 h. The electrical conductivity (L1) was measured, and the leaves (discs) were then autoclaved for 20 min at 120 °C to estimate electrical conductivity (L2). The percent leakage of the electrolytes was measured by using the following formula [46].

(4) $$\mathrm{EL}=\frac{L1}{L2}\;\times \;100$$

### 2.9. Lignin Concentration in the Root

Root lignin was determined by following the well-established protocol [47]. For this purpose, 1 g of sunflower roots were digested in 72% H_2_SO_4_ at 47 °C by vigorously shaking the mixture for 7 min. The partially digested samples were then autoclaved at 121 °C for 30 min for complete digestion. The autoclaved samples were fractionated into soluble and insoluble lignin through filtration. The soluble lignin (in filtrate) was determined by taking OD at 280 nm and 215 nm against the 72% H_2_SO_4_ blank. Lignin was calculated by the following formula:

(5) $$S=\frac{4.53\left(A215\;-\;A280\right)}{300}$$

The equation was generated from these two formulae: A215 = 0.15 F + 70 S and A280 = 0.68 F + 18 S. where, A280 = Optical density at 280 nm, A215 = Optical density at 215 nm, F = The furfural (g), S = The soluble lignin (g).

The 0.68, 0.15, 18, and 70 represent the molar absorptivity of soluble lignin and furfural at 215 and 280 nm, respectively. Insoluble residues were burnt at 550 °C for 4 h, and ash was weighted. The difference in dry mass and ash left after burning were considered as insoluble lignin. The total lignin was determined by adding insoluble and soluble lignin (mg/g of the cell wall).

### 2.10. Visualization of ROS and Their Accumulation in Leaves

For visualization of ROS, the technique of O’Brien et al. [48] was used with minor modifications. Leaves from sunflower seedlings were immersed in the DAB stain solution (2 mL) and kept for incubation at 130 rpm for 4–5 h. To remove the extra DAB stain, the samples were incubated in a bleaching solution (ethanol: acetic acid: glycerol = 3:1:1). To remove chlorophyll, the samples were boiled in ethanol in a water bath, clarified the ROS strained by DAB stain, if any, and then visualized under the light microscope.

### 2.11. Antioxidant System of Sunflower

To assess the antioxidant system of sunflower, the following assays were performed:

#### 2.11.1. 2,2-Diphenyl-1-picrylhydrazyl (DPPH) Radical Scavenging Activity

The DPPH-scavenging activity was determined in the leaves of the plants with minor alteration [49]. Approximately 0.1 g of plant leaves were ground in 1 mL of methanol. A 0.004% methanolic solution of DPPH (Cayman Chemical, Ann Arbor, MI, USA) was also prepared. Initially, a DPPH solution (1 mL) was mixed with the plant sample (0.5 mL), and the mixture was left at room temperature in the dark for 30 min. A decline in color was noted at 517 nm, and the radical scavenging activity was estimated using the following equation:

(6) $$\%\mathrm{DPPH}=\frac{\left(1\;-\;\mathrm{AE}\right)}{\mathrm{AD}}\;\times \;100$$

where AE = Optical density of the mixture of DPPH and leaves extract. AD = Optical density of DPPH solution only.

#### 2.11.2. Catalase and Ascorbate Peroxidases Activity Determination

Catalases (CAT) activity was evaluated by computing the primary rate of H_2_O_2_ cleavage [50]. To 0.1 mL of supernatant, 2.6 mL of 0.05 M PBS (pH 7), 0.1 mM EDTA (ChemCeed, Chippewa Falls, WI USA) and 400 µL of 3% H_2_O_2_ (Univar Solutions, Chicago, IL, USA) were added. Decay in H_2_O_2_ was recorded at 240 nm and expressed as the decay of μM H_2_O_2_ min^−1^.

The protocol of Asada et al. [51] was used to estimate ascorbate peroxidases (APX) activity. The reaction mix contained 0.2 mL of leaves extract, 600 µL of PBS (50 mM, pH 7.0), 100 µL ascorbic acid (0.5 mM) (Sigma Aldrich Burlington, MA, USA), and 100 µL hydrogen peroxide (H_2_O_2,_ 0.1 mM). Measurement of activity was noted at 290 nm and presented as unit min^-1^ mg^−1^ protein.

#### 2.11.3. Peroxidase and Superoxide Dismutase Activity

For the measurement of peroxidase activity, guaiacol was used as a substrate for dehydrogenation [44]. Extraction of enzyme was performed in 3 mL of 100 mM PBS (pH 7.0). Fresh seedling leaves (100 mg) were crushed in 1 mL PBS and swirled for 15 min at 5 °C and 8064 rcf. To 0.1 mL of plant extract, 3 mL PBS (100 mM), 0.05 mL guaiacol (20 mM) (Parchem Fine & Specialty Chemicals, New Rochelle, NY USA) and 0.03 mL H_2_O_2_ (12.3 mM or 0.04%) were added and vortexed. The change in optical density by 0.1 (t) was recorded at 436 nm, and POD activity was calculated by the following formula.

(7) $$\mathrm{Enzyme}\;\mathrm{activity}\;=\;(\frac{500}{∆t})\;\times \;(\frac{1}{1000})\;\times \;(\frac{\mathrm{TV}}{\mathrm{VU}})\;\times \;(\frac{1}{\left(f\;\mathrm{wt}\right)})$$

where; Δt = change in time; TV = total prepared volume and VU = volume of the sample used; f wt = fresh weight (g).

Extraction of SOD was performed with the buffer that consisted of 50 mM KH_2_PO_4_, pH 7.8 and 100 µM EDTA, 1% Triton X-100, 2% Polyvinylpyrrolidone, and a complete protease inhibitor cocktail (Roche, Mannheim, Germany). For this, 100 mg of leaves were homogenized in the buffer mentioned above, followed by sonication (2 × 30 s, at A = 30) (Model; LeelaSonic-50) at 4 °C and filtration (polycarbonate filters, 2.0 µm pore size; Osmonics, South Miami, FL, USA). The filtered sample was centrifuged for 20 min (10,000 rcf) at 4 °C, and the supernatant was carefully collected. The resultant supernatant was used to quantify SOD activity [52].

The activity was assessed by measuring the inhibition in the photoreduction of NBT (nitro-blue tetrazolium) by SOD [53]. The final sample composed of 50 mM PBS (pH 7.6), 100 µM EDTA, 50 mM sodium carbonate, 50 μM NBT (Sigma-Aldrich, Burlington, MA, USA), 10 μM riboflavin (BIOCHEM Bernburg GmbH, Bernburg Germany), 12 mM L methionine (Fagron Industry, Rotterdam, ZUID-HOLLAND Netherlands), and 100 μL crude extract at a final volume of 3 mL. Blank for the sample consist of all the mentioned chemical without leaves extract. Exposure to white light for 15 min initiated the reaction. Following incubation, optical density was measured at 560 nm. A unit (U) of SOD is characterized as the unit of enzyme necessary to prevent NBT from degrading photochemically by 50%.

#### 2.11.4. Estimation of Reduced Glutathione

Reduced glutathione (GSH) (Chambio, Shanghai, China) was estimated by measuring the redox reaction of NADPH (Sigma-Aldrich, Burlington, MA, USA). The reaction mixture consisted of 0.3 mL leaves extract, 1.8 mL PBS, 0.3 mL of EDTA, 0.3 mL of NADPH, and 0.3 mL of oxidized glutathione (GSSG) (Chambio, Shanghai, China). The optical density was recorded at 340 nm [54].

### 2.12. Estimation of Heavy Metals

#### 2.12.1. Colorimetric Determination as Preliminary Test

The bacterial strain and plants were grown as mentioned above in the concentration of Cr^+6^. The isolate P1 was inoculated in the sterilized broth media and incubated at 28.2 °C for 24 h. The supernatant and pellet were isolated by centrifugation for 10 min at 5600 rcf and 4 °C. The quantity of Cr was then determined in the supernatant. Supernatant from the bacterial culture was directly subject to Cr^+6^ estimation using the di-phenyl carbazide technique; however, plants samples were exposed to wet digestion to determine Cr^+6^ following the same protocol [55].

#### 2.12.2. BCR Sequential Extraction Method

The technique of the Community Bureau of References (BCR) was used to estimate the two different species of chromium [56]. The extraction involved three main stages:

##### Stage 1: Exchangeable or Acid-Soluble

To the dried plant sample (0.5 g or 0.5 mL bacterial culture/exudates), 20 mL of acetic acid (110 mM) (Sigma Aldrich, Burlington, MA, USA) was mixed, kept in a shaker for 12–24 h at 25–30 °C, and separated from the residues through centrifugation at 504 rcf at 28 °C for 3 min.

##### Stage 2: Cr^+6^ Extraction

The remains obtained in stage 1 were added to a freshly prepared 20 mL solution of 500 mM hydroxylamine-hydrochloride (Sigma Aldrich, Burlington, MA, USA), and pH 1.5 was adjusted with nitric acid (Sigma Aldrich, Burlington, MA, USA). The resultant solution was incubated at 25–30 °C for 16 h. The residues were separated via centrifugation, as mentioned in step1.

##### Stage 3: Cr^+3^ Extraction

The remains of stage 2 were treated with 30% hydrogen peroxide (5 mL) 2 times and incubated for 1 h (pH set to 2), followed by drying. To the dried samples, 1 M ammonium acetate (25 mL) was added and kept for incubation at 28 °C for 16 h.

The pellets were washed at each step of the extraction, shaken (15 min), and centrifuged (20 min) at 504 rcf. The supernatant was deliberately washed away, avoiding contamination from the residues. This procedure was repeated to eliminate any residual reactants and Cr from the preceding level.

#### 2.12.3. Atomic Absorption Spectroscopy (AAS)

Isolated portions of the above steps were examined via atomic absorption, Perkin Elmer (AAnalyst 700, Parkin Elmer, Waltham, MA, USA). The air/acetylene flame was used to perform the measurements under the operational parameters recommended by the manufacturer.

### 2.13. Bioconcentration Factor

The concentrations of Cr in the seedling biomass were calculated using the following equations:

(8) $$\mathrm{BCF}\;=\;\frac{\mathrm{Cr}\;\mathrm{in}\;\mathrm{Plant}\;\mathrm{biomass}}{\mathrm{Cr}\;\mathrm{added}\;\mathrm{to}\;\mathrm{media}}$$

### 2.14. P1 Colonization with Host Roots

The plate count technique was followed to test the colonization of selected isolate with host roots. For this, 2 cm of host root was forcefully shaken to detach loosely attached bacteria in the sterile distilled water and then crushed into 1 mL of 10 mM MgCl_2_ solution. The resulted mixture was filtered and serially diluted up to 10^−6^, and 0.1 mL of each was plated on LB agar. The colonies were counted after 24 h of incubation at 28 °C.

### 2.15. Data Analysis

All the assays were carried out three times, and the results of the experiment were categorized into Cr^+6^ and P1 inoculations. The significance (*p* < 0.05) for *A. bouvetii* inoculation status, Cr levels, and their interaction were determined using ANOVA and Duncan’s Multiple Range Test (DMRT) in IBM SPSS Statistics 21(IBM, Armonk, NY, USA). Graph Pad Prism (Version 5.03, Graph Pad Inc., San Diego, CA, USA) was used for plotting graphs.

## 3. Results

### 3.1. P1 Molecular Identification

**Figure 1:**
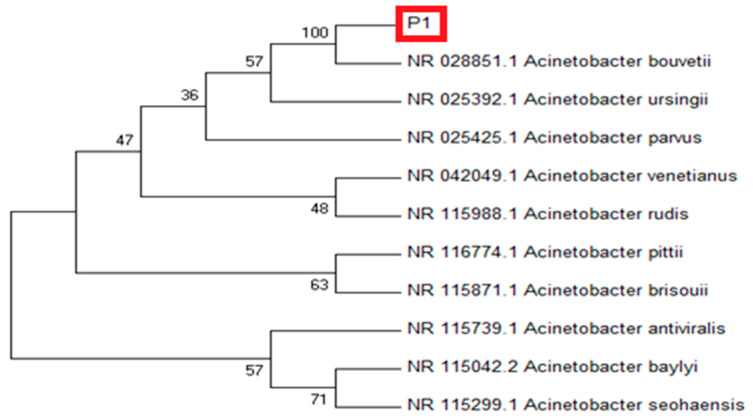
Molecular phylogenetic analysis of rhizobacterial strain P1 (red frame) showing evolutionary relationships of taxa using NJ (Neighbor Joining) tree method. The analysis of the evolutionary relationship was carried out in MEGA7 using 16 S rDNA sequences of our isolate and closely related sequences retrieved from NCBI GenBank. Nodes are labeled with bootstrap values.

Based on the 16S rRNA sequence, the isolate P1 showed maximum sequence homology (97.17%) with *Acinetobacter bouvetii* (Figure 1). For confirmation, the sequence was subjected to phylogenetic analysis by constructing their phylogenetic consensus by Neighbor Joining (NJ) using MEGA 7. The isolate P1 showed a close resemblance with *A. bouvetii* supported by 100% bootstrap value in the tree. In short, the strain P1 was identified as *A. bouvetii* through BLAST results and the phylogenetic analysis. The obtained sequence can be retrieved through a GenBank accession No. MT478039 on NCBI nucleotide database.

### 3.2. Characterization of the Selected Strains

#### 3.2.1. Impact of Chromate Rhizobacterial Growth

The rhizobacterial isolate P1 was allowed to grow in L.B. broth supplemented with different concentrations of Cr^+6^. An increase in bacterial growth was recorded in an L.B. broth supplemented with 100 µg/mL of Cr^+6^. The steady growth of rhizobacterial isolate P1 was recorded in L.B. broth supplemented with 300 µg/mL of Cr^+6^ (Figure S1). An approximately 3-fold increase was recorded in bacterial growth at 500 µg/mL supplementation of broth with Cr^+6^. However, a decline was recorded in the growth of the rhizobacterial strain P1 as the concentration of Cr^+6^ was increased in broth. Interestingly, the recorded growth of the bacteria was higher compared with control at all supplemented levels of Cr^+6^.

#### 3.2.2. Production of Bioactive Compounds

**Figure 2:**
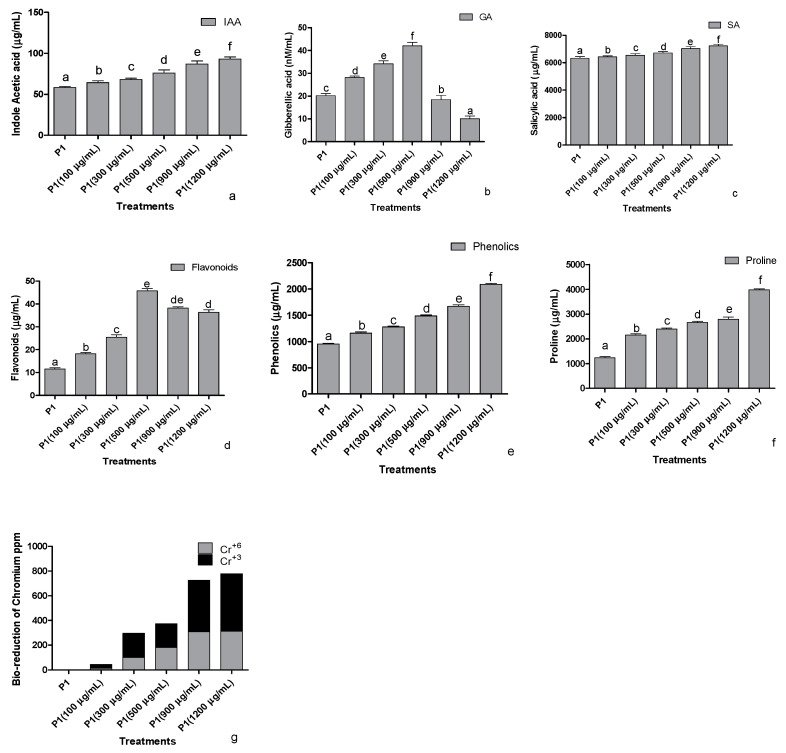
Effect of chromate stress on the concentration of (**a**) indole acetic acid, (**b**) gibberellic acid, (**c**) salicylic acid, (**d**) flavonoids, (**e**) phenolics, and (**f**) proline in the bacterial culture supernatant and (**g**) bioreduction capability of the rhizobacterium P1. The rhizobacterium was grown in the LB broth containing different quantities of hexavalent chromium for 24 h, and the supernatant was then assessed for the mentioned parameters. Data are means of nine replicates with ±SE of the mean. Different alphabets represent significance (*p* < 0.05).

The strain P1 was screened against the different concentrations of K_2_CrO_4_ (Potassium chromate) as a Cr^+6^ source to assess the indole acetic acid production in stressed conditions (Figure 2a). The addition of Cr^+6^ stimulated the rhizobacteria to produce elevated levels of IAA in a concentration-based manner. The release of gibberellic acid (GA_3_) was also improved as the concentration of the Cr^+6^ increased. A significant concentration-dependent increase in the production of GA_3_ was noted at 500 µg mL^−1^ of the Cr^+6^, however, beyond 500 µg mL^−1^ of stress, the production of the GA_3_ decreased (Figure 2b). A similar pattern was also recorded in the production of salicylic acid upon the induction of stress by the concentrations of Cr^+6^ (Figure 2c). Bacterial salicylic acid production increased with the elevation of metal levels supplemented from 100 to 1200 µg mL^−1^ of Cr^+6^ in the media.

Significant impacts were recorded on total flavonoids production by P1 at an elevated concentration of the Cr^+6^ (Figure 2d). An increasing tendency in the release of total flavonoids was noted from 0 to 500 µg mL^−1^ of the metal supplemented in media; however, a dip was noted in the flavonoid concentration of the strain upon exposure to 900 µg mL^−1^ or 1200 µg mL^−1^ of the Cr^+6^. Higher production of phenols in Cr^+6^ spiked media was observed (Figure 2e). Further production was positively regulated with higher levels of chromate-induced stress peaking at 1200 µg mL^−1^. Proline produced by the selected strain was determined after exposing the strain to various concentrations of the Cr^+6^ (Figure 2f). The proline concentrations showed an abrupt increase at 100 µg mL^−1^ of Cr^+6^, which steadied till 900 µg mL^−1^ of Cr^+6^. Elevated proline levels were recorded at 1200 µg mL^−1^, whereas the lowest was recorded in the growth medium supplemented with 0 µg mL^−1^ of Cr^+6^.

Beyond the production of plant regulators and stress mitigators, the selected rhizobacteria had the potential for efficient solubilization of the inorganic phosphate. The phosphate solubilization index of the strain was 3.66.

#### 3.2.3. Reduction of Cr^+6^ to Cr^+3^

When the selected strains were exposed to 100, 300, 500, 900, and 1200 µg mL^−1^ of the Cr^+6^, the strains were able to reduce the valency of Cr^+6^ and biotransform it into their least toxic form Cr^+3^ (Figure 2g). After 24 h of treatment, the strain biotransformed almost half of the highly toxic Cr^+6^ to its least toxic, trivalent form.

### 3.3. Net Assimilation Rate (NAR) and Relative Growth Rate (RGR)

**Figure 3:**
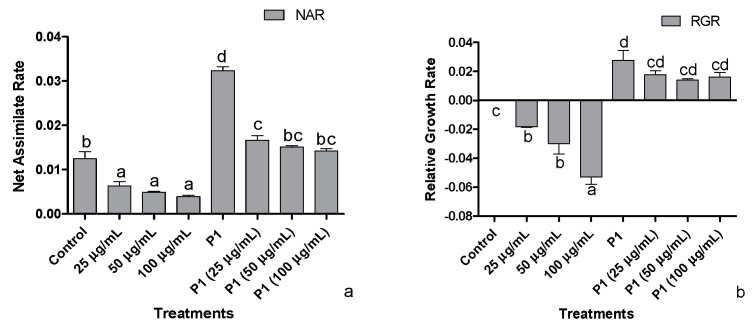
Effects of different concentrations of hexavalent chromium and P1 on (**a**) net assimilation rate and (**b**) relative growth rate of sunflower seedlings grown hydroponically (half-strength Hoagland’s solution) for 16 days in a plant growth chamber. Data are mean of 36 replicates with SE of the mean. Different labels on mean bars represent significance (Duncan; *p* < 0.05).

Decreasing trends were observed in the host’s net assimilation rate, i.e., showing an inverse proportion to increasing metal concentration (Figure 3a). With the plant growth promoting activities of P1, the net assimilation rate of the host was positively regulated exhibiting an increase in net assimilation rate under Cr^+6^ induced stressed environment. Plant growth promoting the interaction of P1 with their host achieved the maximum net assimilation rate related to normal and stressed control plants and all other treatments.

The dip in relative growth rate was roughly 5-folds in the host treated with 100 µg mL^−1^ of Cr^+6^ (Figure 3b). With inoculation of P1, higher RGR was achieved by all host plants grown in Hoagland’s media supplemented with the stated level of Cr^+6^. The highest RGR was approximately 3-fold the level exhibited by the host associated with P1. Nonetheless, the decline was recorded with the metal concentration, but the RGR was still higher than normal and stressed control plants, i.e., RGR of P1 associated plants exposed to 100 µg mL^−1^ of Cr^+6^ was approximately two times that of the non-associated control plants.

### 3.4. Effects of P1 and Chromate Stress on Metabolites of Host Plants

Endogenous and root exudates indole acetic acid (IAA) of the chromate stressed *H. annuus* seedlings was assessed (Table S1). Inverse relation of Cr^+6^ was recorded with the endogenous and exogenous IAA levels in *H. annuus* seedlings. Inoculation of seedlings with P1 benefited the host plant by significantly improving IAA production and its exudation through the root. Different levels of chromate significantly reduced the amount of endogenous and released IAA in P1 associated seedlings, but its concentration was still higher than the seedlings grown in the absence of Pa strain.

Exposure of *H. annuus* seedlings to Cr^+6^ significantly enhanced the concentration of plant and root exuded flavonoids in a dose-dependent manner (Table S1). Inoculation of the seedlings with the strain P1 controlled the accumulation of the endogenous flavonoid’s concentration keeping their levels below the noninoculated counterparts. However, P1 associated plants released significantly greater quantities of flavonoids which were synergistically enhanced upon exposure to chromate stress.

Under chromate stress, sunflower seedlings had a significantly lower concentration of total phenols in plants and in root exudates than the control seedlings (Table S1). Chromate concentration was inversely related to the phenolic concentration in the seedlings as well as in the exudates. Inoculation of *H. annuus* seedling P1 significantly enhanced the concentration of endogenous and released phenols than the control seedlings. A further increase was noted in the endogenous phenols of P1 associated sunflower seedlings exposed to chromate in a concentration-dependent manner. However, chromate-stressed P1 inoculated seedlings had a lower number of released phenols than the P1 seedlings grown in the absence of chromate stress.

Proline accumulation and release were also enhanced in the chromate-stressed seedlings than in control (Table S1). Its endogenous concentration was further enhanced in P1 inoculated seedlings, and the exposure of these seedlings to chromate caused a reduction in proline concentration. The concentration of released proline was greater in P1 inoculated seedlings than in the control seedlings. In the presence of chromate, such seedings released even higher concentrations of proline.

### 3.5. Root lignification, Electrolyte Leakage, and Malonaldehyde Concentration

**Figure 4:**
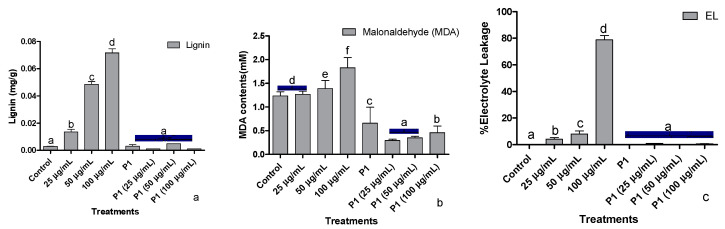
Effect of different concentrations of hexavalent chromium and P1 on (**a**) root lignification, (**b**) leaf malonaldehyde, and (**c**) electrolyte leakage of sunflower seedlings grown hydroponically (half-strength Hoagland’s solution) for 16 days in a plant growth chamber. Data are mean of 36 replicates with SE of the mean. Different labels on mean bars represent significance (Duncan; *p* < 0.05).

Stressful conditions due to elevated levels of chromate upregulate the lignin biosynthesis, thereby increasing lignin deposition. An almost two times increase in lignin deposition was recorded at each increase in metal concentration (Figure 4a). Interestingly, a dip in lignin production at all treated concentrations of the metal was noted after P1 inoculation.

Exposure of sunflower seedlings to hexavalent chromium enhanced the accumulation of malondialdehyde (MDA). An increase in the endogenous concentration of MDA was dependent on the amount of chromate present in the growth medium (Figure 4b). Rhizobacterium P1 colonized seedlings had a significantly lower concentration of MDA, which was further reduced upon exposure to chromate.

Upon exposure to the said levels of Cr^+6^, sunflower seedlings showed a concentration-based increase in the leakage of electrolytes. The electrolyte leakage doubled at each elevated level of Cr^+6^ (Figure 4c). The host seedlings were rescued by P1 inoculation, which reduced electrolytes leakage from sunflower leaves by several folds. Even in the presence of 100 µg/mL of chromate, electrolyte leakage from the leaves of P1 associated seedlings was lower than the control seedlings.

### 3.6. Antioxidant

**Figure 5:**
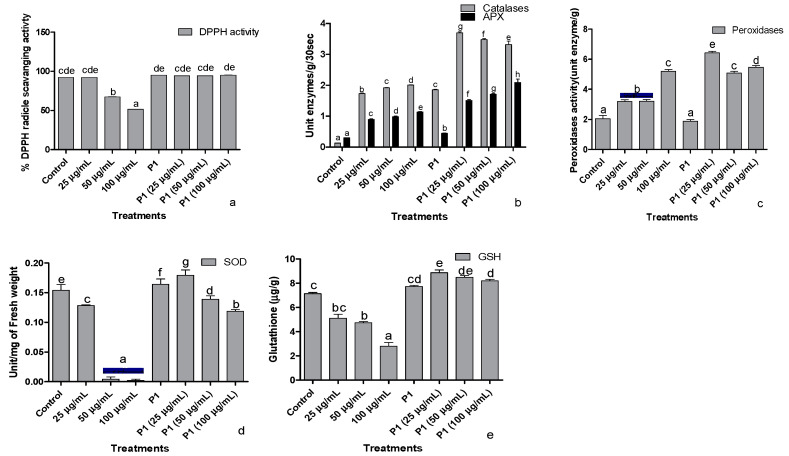
Effect of different concentrations of hexavalent chromium and P1 on (**a**) %DPPH radical scavenging activity, (**b**) catalases and ascorbate peroxidase, (**c**) peroxidase, (**d**) superoxide dismutase, and (**e**) reduced glutathione of sunflower seedlings grown hydroponically (half-strength Hoagland’s solution) for 16 days in a plant growth chamber. Data are mean of 36 replicates with SE of the mean. Different labels on mean bars represent significance (Duncan; *p* < 0.05).

The ability of seedling’s leaf extract to scavenge DPPH free radical dropped significantly in the presence of 50 and 100 µg/mL of hexavalent chromium (Figure 5a). The lowest DPPH scavenging activity was noticed in the host plants treated with 100 µg mL^−1^ of Cr+6. However, rhizobacterium P1 Inoculated seedlings showed a stable DPPH free radical scavenging activity even in the presence of the highest concentration of chromate used in this study.

Ascorbate peroxidases and catalase activities were enhanced when sunflower seedlings were exposed to different concentrations of chromate (Figure 5b). The activity of both the enzymes increased with the increasing concentration of hexavalent chromium in the media. In P1 inoculated seedlings, activities of catalase and peroxidase were significantly greater than the control seedlings. Upon exposure to 25 µg mL^−1^ chromate, the activities of these enzymes increase by 2-fold. The rise in APX activity continued with an increase in chromate concentration. However, further increases in chromate concentration significantly reduced catalase activity in sunflower seedlings.

Exposure to chromate also induced peroxidase activity in sunflower seedlings in a concentration-dependent manner (Figure 5c). In P1 inoculated seedlings, peroxidase activity did not show any difference in comparison to the control seedlings. However, P1 associated seedlings had significantly higher peroxidase activity upon exposure to different concentrations of chromate than their respective controls.

Exposure of sunflower seedlings to hexavalent chromium was associated with reduced SOD activity than the control. Higher concentrations (50 and 100 µg mL^−1^) more severely affected the activity of this enzyme which was reduced by several folds (Figure 5d). The rhizobacterium P1 enhanced SOD activity than its level in the noninoculated seedlings. The strain also reduced the harmful effect of chromate on SOD activity.

The concentration of reduced glutathione also declined in chromate-treated seedlings, but the effect was moderate compared with SOD activity (Figure 5e). Inoculation of seedlings with P1 significantly enhanced the concentration of reduced glutathione in the host plant compared to the control seedlings. Exposure of P1 associated seedlings either further improved the concentration of reduced glutathione or did not influence its level at all.

### 3.7. 3,3′-Diaminobenzidine (DAB) Stain Assay

**Figure 6:**
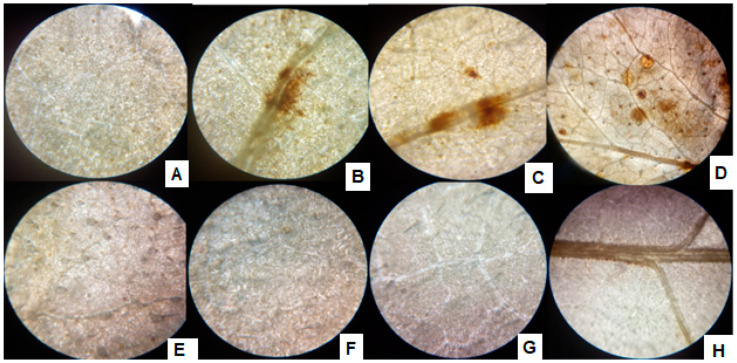
ROS accumulation in leaves of P1 inoculated sunflower seedlings exposed to different levels of hexavalent chromium (**A**) control (0 µg mL^−1^), (**B**) 25 µg mL^−1^, (**C**) 50 µg mL^−1^, (**D**) 100 µg mL^−1^, and (**E**) P1 strain + 0 µg mL^−1^, (**F**) P1 + 25 µg mL^−1^, (**G**) P1 + 50 µg mL^−1^, and (**H**) P1 + 100 µg mL^−1^. The seedlings were grown hydroponically (in half-strength Hoagland’s solution) for 16 days in a plant growth chamber. Fully expanded leaves were detached and stained with DAB to visualize ROS.

With the exposure of sunflower seedlings, the H_2_O_2_ production was noted as brown spots formed by DAB strain in the leaves, and with the increase of Cr^+6^ supplementation, the size and number of brown spots increased (Figure 6). The inoculation of the P1, however, alleviated the Cr^+6^ induced stress and hence reduced the H_2_O_2_ production, which is recognized by spotless tissues after treatment with DAB strain.

### 3.8. Determination of Uptake and Accumulation of Cr^+6^ by Host Plants

#### 3.8.1. By Colorimetric Method

**Figure 7:**
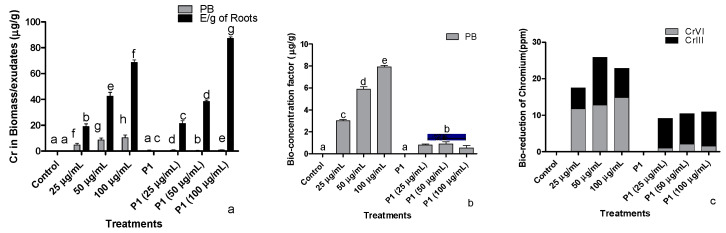
Influence of rhizobacterium P1 on (**a**) absorptions/accumulation, (**b**) bioconcentration, and (**c**) bio-reduction of Cr^+6^ in sunflower seedlings. The seedlings were grown hydroponically in Hoagland media (half strength) containing different concentrations of hexavalent chromium for 16 days in a plant growth chamber. Data are a mean of 36 replicates with SE and the alphabets representing significance (Duncan; *p* < 0.05).

The amount of Cr^+6^ accumulated by different parts of sunflower seedlings increased with an increase in the concentration of the Cr^+6^ in the medium (Figure 7a). For instance, the seedling exposed to 100 µg mL^−1^ accumulated about 10.34 µg mL^−1^ of hexavalent Cr^+6^ in their parts, which was the highest among the plants treated with various concentrations of Cr^+6^. Accumulation of heavy metals in plant parts was significantly reduced in seedlings associated with P1.

#### 3.8.2. Bioconcentration of Cr^+6^ in Host

Sunflower seedlings exposed to increased concentration of Cr^+6^ showed an increase in bioaccumulation of Cr^+6^ (Figure 7b). Conversely, the inoculation of *H. annuus* seedlings with rhizobacterial strain P1 inhibited the accumulation of Cr^+6^ as the concentration increased in the medium to avoid phytotoxicity. The ceased accumulation of Cr^+6^ in P1 associated *H. annuus* seedlings were noted at all treated level of Cr^+6^.

#### 3.8.3. Determination of Cr Species by BCR Extraction

Exposure of *H. annuus* seedlings to various concentrations of the Cr^+6^ showed higher accumulation in all parts of the plant, where a small amount has been converted to a stable Cr^+3^ form. Indeed, the bioaccumulation of Cr^+6^ increased when applied at higher concentrations (Figure 7c). With inoculating P1, a reduction in the bioaccumulation of Cr^+6^ was recorded in all parts of the host plant. Moreover, the highest percent conversion of Cr^+6^ to a nontoxic form (Cr^+3^) was noted in the plant biomass treated with rhizobacterial strain P1.

#### 3.8.4. Assessing Root Colonization Potential of P1

P1 shows an increase in the root colonizing capacity as the supplements of the Cr^+6^ in the medium increase. The root colonization tendency from strain control to treatments, i.e., 25 µg/mL 50 µg/mL and 100 µg/mL were 4.6 × 10^8^, 5.0 × 10^8^, 1.18 × 10^9^, and 1.30 × 10^9^ bacteria, respectively, in 2 cm of root segment.

## 4. Discussion

The rhizobacterial isolate P1 was able to tolerate a concentration of chromate up to 12,000 times higher than the WHO permissible levels (0.1 mg L^−1^) [57]. A significant increase was recorded in the growth of bacterial mass at all the applied levels of Cr^+6^ in the liquid medium. The rhizobacterium was also capable of secreting substantial quantities of phytohormones, including IAA, GA, and SA, demonstrating its pro-plant nature [33,58]. We noticed that increased stress levels enabled the strain to release higher amounts of these phytochemicals. In fact, higher production of these phytohormones by P1 under elevated stress levels is an important outcome that can benefit the host plant to withstand the stress [36]. The previous findings suggest that plant species have the ability to absorb phytohormones released by the microbes in the rhizosphere [59]. Moreover, the rhizobacterium was able to colonize sunflower roots where it can directly contribute to phytohormones and improve the host fitness and growth. Sunflower seedlings colonized by the rhizobacterium P1 had comparatively higher endogenous levels of these phytohormones than the non-associated seedlings. Additionally, with the absorption of microbial contributed phytohormones, binding of salicylic acid Calcium-dependent protein Kinases (CDPKs) activating stress-responsive downstream genes (Peroxidases, GSTs (Glutathione S-transferases), Osmotins, HSPs (Heat shot proteins)), and can be used to combat a range of abiotic and biotic stressors in the long term [60]. Increased expression of CDPKs and downstream stress-responsive genes in over-expressed lines relative to knockdown lines indicates *OsMYB-R1* mediates stress tolerance through auxin and salicylic acid-responsive signaling [61]. Among the phytohormones, gibberellin potentially enhances the agronomic attributes of plants. He et al. [62] investigated the significant effect of gibberellic acid in stress acclimation in tested plants by the upregulation of *TaMYB73* gene expression. Hyper-expression of the *Triticum TaMYB73* gene in *A. thaliana* improves the host acclimation potential to NaCl and upregulates the expression of several genes such as *AtCBF3, AtABF3, AtRD29A,* and *AtRD29B*. These are the key factors of osmotic adjustment that influence the crosstalk with microbial phytohormones in the rhizosphere, thus boosting immunity and growth of the host under a stressed environment [61].

Enhanced production of phenolic, flavonoid, and proline in the rhizosphere occurs by the stimulation of stress because they act as nonenzymatic antioxidants and enhance the capability of rhizobacteria to bear metal toxicity [63,64]. Rhizobacteria employ flavonoids to chelate soil nutrients and gain adequate nutrients when they are stressed. More crucially, flavonoids influence quorum sensing, allowing bacteria to carry out their density-dependent tasks [65]. Microbes employ phenolics and flavonoids as cross signals as part of a chemical interaction to form a plant growth-promoting relationship with the host root. Flavonoids, phenols, and proline operate as ROS scavengers inside the cell, a key step in stress tolerance [66,67]. Apart from all these characteristics, the selected rhizobacterial efficiently solubilize inorganic phosphate that helps the rhizobacteria to promote host growth normally in a stressful environment [68]. Recent studies also found that PGPR alleviate metal stress and enhance the agronomic attribute of the host. Moreover, they showed that the height of plants increased by 22–50% after inoculation with SS6, SS1, and SS3 exposed to Cr (20, 30, and 40 ppm) compared with the non-inoculated plants [27,69]. In another study, the application of *P. aeruginosa* strain OSG41, even with three times concentration of chromium, increased the dry matter accumulation, symbiotic attributes (like nodule formation), grain yield, and protein of chickpea compared with non-inoculated plants [30].

Susceptibility of sunflower seedlings was noted at elevated levels of Cr^+6^, exhibiting serious consequences in terms of growth attributes, including low net assimilation rate and relative growth rate (*p* < 0.05) [70]. The decrease was accompanied by a sharp decline in the growth regulators, particularly IAA, GA, and SA, and a malfunctioned antioxidant system, which is necessary for the normal growth and development of the plant species [71]. Inability to produce enough phytohormones left sunflower seedlings at the mercy of chromate toxicity, leading to reduced growth and higher accumulation of ROS. Under such circumstances, the severity of chromate stress was further increased due to loss in the ability of host plant species to scavenge ROS. Recently, the role of IAA was reported to aid in stress acclimation; however, severe metal toxicity resulted in the degradation of IAA [72]. 

In the presence of *Acinetobacter bouvetii* P1, the seedlings had higher NAR and RGR and were more tolerant to chromate stress than the uninoculated seedlings. Sunflower seedlings received the aid of phytohormones from the associated rhizobacterial isolates, which boost the host′s fitness to withstand harsh environments while growing normally. Higher IAA levels are correlated with improved growth, yield, and stress tolerance of the host plants. The same increasing pattern of total flavonoids production was noted with Cr^+6^ elevation. The flavonoids act as metal chelators, help quenching reactive oxygen species and act as a nonenzymatic antioxidant. With the inoculation of P1, a decreasing pattern in the endogenous flavonoids was observed. Nonetheless, a concentration-based increase in the exogenous flavonoids was also recorded. The lower accumulation and higher exudation of flavonoids may be a strategy of the host plant to quench the metal in the rhizosphere to avoid the possible phytotoxicity resulting from metal accumulation in the plant body. In such conditions, flavonoids chelate the toxic metal as well as ROS quenchers to detoxify the oxidative stress produced as a result of an excess of Cr^+6^ [73]. An interesting observation was the severe reduction in the total endogenous and exogenous phenolics with chromate exposure of the host, making it susceptible to various biotic and abiotic stresses [33]. Metal stress disturbs the shikimate pathway resulting in the lower production of total phenolics. The phenolics act as a part of the defense system and detoxify the ROS, working as a nonenzymatic antioxidant, and their decline leads to a susceptibility of the host [74]. Plant growth-promoting associations of P1 improve the phenolic production of the hosts and help to detoxify the metal, and subsequent oxidative impairment in the host, acclimating the host to metal toxicity and subsequent oxidative damage [75].

Higher proline production is the key response to a stressful environment acting as an osmolyte and nonenzymatic antioxidant to minimize the toxicity caused by the stressor. Their endogenous and exogenous production was further improved with the application of P1. The endogenous proline accumulation in the cell tends to protect the oxidative damage and metal toxicity whereas, the increased exogenous proline tends to chelate metal in the rhizosphere, thereby avoiding the uptake of the metal and preventing phytotoxicity and subsequent oxidative damage [76,77].

In some instances, higher lignification is a tendency of the host to develop a physical barrier to avoid the entry of such toxic substances. However, early hyper-lignification due to abiotic stressors results in stunted growth in plants [78]. A recently published review [79] suggested that hyper-lignification of the plant exposed to Cd stress sufficiently reduced the entry of Cd into the plant’s cell. Another study found that, since Cd is primarily adsorbed at the root level, the anatomy and molecular structure of the cell wall in root cells is a critical parameter. Plant roots rich in suberin and lignin may be more impermeable to Cd and therefore offer resistance to Cd absorption and translocation [80]. The same tendency of root lignification was noticed in sunflower seedlings challenged with Cr^+6^. However, the degree of lignification was not enough to completely exclude Cr^+6^ from the roots. The rhizobacterium-associated seedlings had limited access to Cr^+6^, bringing lignification to a normal level that further promotes normal cell division and elongation [81,82]. In short, the P1 isolate helped the host plant restrict the entry of Cr^+6^. P1 also contributed towards proline production to alleviate the stress and led to bioreduction of Cr^+6^, strengthened antioxidant system of the host, improved host endogenous phytohormones pool, and solubilized nutrients (phosphorous).

Apart from growth-promoting activities, P1 also interfered with the seedlings’ metal uptake, contributing to a 90–95% decrease in seedlings’ Cr absorption. The ceased absorption of Cr^+6^ by root in Cr^+6^ supplemented Hoagland’s medium may be attributable to the capacity of P1 to biotransform the highly toxic Cr^+6^ form to their least toxic Cr^+3^ form in the host’s rhizosphere [83]. The application of *P. aeruginosa* strain OSG41 improved the dry matter accumulation, symbiotic features (such nodule formation), grain production, and protein contents of chickpea exposed to Cr^+6^ stress. The bioinoculant reduced Cr^+6^ absorption by 36, 38, and 40% in roots, shoots, and grains, respectively [30]. In addition to a bioreduction in the rhizosphere, chromate reduction in plants also tended to occur with isolate P1. Bioreduction of heavy metal is one of many PGPR approaches to counteract its toxic impact because Cr^+3^ is an active human oligo-element [84,85,86,87]. Also, Cr^+3^ is relatively less toxic, chiefly to the crops, and at small quantities (0.05 mg L^−1^), it may encourage growth and production [88,89].

The capacity of P1 to enhance the vegetative attributes of sunflower seedlings stressed by Cr^+6^ implied that chromate detoxification and limited absorption are not the predominant phytostimulation process. Moreover, increased storage of enzymatic antioxidants (CAT, APX, POD, and SOD) along with reduced glutathione (nonenzymatic) may have scavenged the generated ROS in plants more efficiently to support their defensive mechanisms [90]. Evidence of improved ROS scavenging ability of rhizobacterium associated sunflower seedlings was linked to the effective DPPH quenching potential and reduced ROS accumulation. In plants, oxidative pressure can be recognized by enhanced production of ROS under stress conditions due to lower production of several antioxidant enzymes and higher malonaldehyde concentration [91,92] causing membrane disruption and hence leakage of essential electrolytes [93]. Therefore, the rhizobacterial isolate P1 in the current scenario has supported the *H. annuus* in releasing significant amounts of enzymatic and nonenzymatic antioxidants to detoxify the accumulated ROS and cease the MDA production thus, preventing electrolyte leakage in stress conditions. Within unhealthy environments, the activation of these enzymes scavenged the ROS, thereby enabling the plant to grow healthy. The bacterial strain either contributes to, or triggers, the host to produce substantial quantities of antioxidants. Liu et al. [94] unveiled the RNA-seq data that FZB42 (a PGPR) triggered the overexpression of photosynthesis-related genes, improved ROS scavenging machinery, osmoprotectants (trehalose and proline), Na1 translocation as well as jasmonic acid, auxin, and ethylene signaling in salt stress conditions. In another experiment, Bharti et al. [95] presented that *Dietzia natronolimnaea* STR1 inoculated salt-stressed wheat plants expressed the *TaWRKY10* gene. The *WRKY* TFs have indeed a significant role in stress mitigation by adjusting the osmotic balance of cells, ROS detoxification processes, and regulation of stress-related genes. The enhanced activities of defense-related enzymes contributed to the bioprotection of plants against various biotic and abiotic stress factors. This enhanced antioxidant production induces systematic resistance in the host plants.

In addition to the generation of antioxidants, colonizing roots of the host, and minimizing metal uptake, the rhizobacteria P1 often releases proline on its own to facilitate the host proline production and cope with elevated levels of metal stress [96]. Also, the strain ably converted the Cr from an extremely toxic form (Cr^+6^) to a relatively less toxic form (Cr^+3^), thus decreasing its entrance into the food chain and protecting the humans from the toxic effects of chromate.

## 5. Conclusions

From the study, it is concluded that the use of plant growth-promoting rhizobacteria P1 is a sustainable way to improve host growth under Cr^+6^ stress. The stress-induced growth reduction is accompanied by a decline in the endogenous pool of phytohormones and a compromised antioxidant system reflected by enhanced accumulation of ROS. However, the rhizobacterium *A. bouvetii* P1 association with sunflower seeds under Cr^+6^ stress enables the host to withstand up to 1200 µg/mL of Cr^+6^ without compromising its growth. Such seedlings have enough phytohormones and improved antioxidants (enzymatic and nonenzymatic) to scavenge the ROS generated during Cr^+6^ stress. Mobilization of nutrients, such as phosphate by the associated rhizobacterium, also supports the plant’s better performance. Furthermore, P1 reduces Cr toxicity by making it unavailable to the plants, and in doing so, P1 removes the Cr^+6^ from the food chain, protecting humans from the toxic effects of Cr^+6^. With all this in mind, *A. bouvetii* can be used as a potent strain in field trials instead of synthetic fertilizers that subsequently lead to soil deterioration.

## Data Availability

All the data are included in the manuscript.

## Supplementary Materials

The following are available online at https://www.mdpi.com/article/10.3390/antiox10121868/s1, Figure S1: Effects of different chromium levels of bacterial growth. Data represent mean with SE ± and letter represent significance (*p* < 0.05); Table S1: Effect of Cr^+6^ and P1 on indole acetic acid contents, total flavonoids, total phenolics, and Proline contents in plant biomass (PB) and their exudation by root (E/g of Roots).
