# Supplementary material for: Phytohormones Producing Acinetobacter bouvetii P1 Mitigates Chromate Stress in Sunflower by Provoking Host Antioxidant Response
Source: Antioxidants (Basel). 2021 Nov 24;10(12):1868. doi: 10.3390/antiox10121868 (PMC8698644; doi:10.3390/antiox10121868)
Supplement: Supplementary file 1 [file antioxidants-10-01868-s001.zip › antioxidants-1416367-supplementary.pdf]

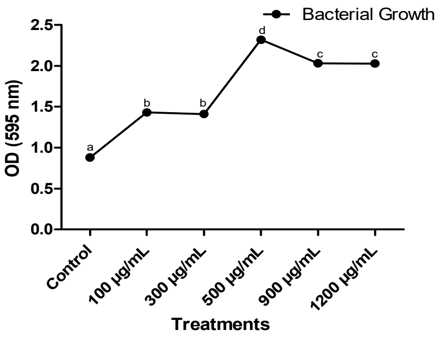

Figure S1. Effects of different chromium levels on the bacterial growth. Data represent mean with SE± and letter represent significance ($p < 0.05$).

Table S1. Effect of Cr^{+6} and P1 on indole acetic acid contents, total flavonoids, total phenolics and Proline contents in plant biomass (PB) and their exudation by root (E/g of Roots). Different alphabets represent significance ($p < 0.05$).

**Treatment**	**Indole Acetic Acid (µg/g)**		**Total Flavonoids (µg/g)**		**Total Phenolic (µg/g)**		**Proline contents (mg/g)**	
	**PB**	**E/g of Roots**	**PB**	**E/g of Roots**	**PB**	**E/g of Roots**	**PB**	**E/g of Roots**
Control	69.28^{d} ± 1.17	12.84^{e} ± 0.64	10.91^{d} ± 0.31	3.76^{a} ± 1.17	526.6^{d} ± 1.17	41.33^{d} ± 0.1	0.75^{a} ± 0.007	0.52^{a} ± 0.001
25 ugmL^{-1}	46.30^{c} ± 0.28	11.08^{c} ± 0.87	11.95^{e} ± 0.54	5.98^{b} ± 0.84	510.4^{c} ± 2.28	5.12^{c} ± 0.84	0.84^{b} ± 0.002	0.56^{b} ± 0.008
50 ugmL^{-1}	10.82^{a} ± 1.56	9.80^{b} ± 0.56	13.72^{g} ± 1.06	8.00^{d} ± 0.21	457.9^{b} ± 1.56	0.41^{a} ± 0.32	1.02^{c} ± 0.01	0.74^{e} ± 0.003
100 ugmL^{-1}	12.34^{b} ± 0.89	9.21^{a} ± 1.38	14.33^{h} ± 1.06	7.94^{c} ± 1.06	409.2^{a} ± 1.89	3.05^{b} ± 0.11	1.67^{d} ± 0.01	1.17^{f} ± 0.001
P1	82.19^{h} ± 0.71	12.13^{d} ± 1.84	8.31^{a} ± 0.54	23.00^{e} ± 0.76	593.7^{e} ± 2.71	60.76^{h} ± 0.76	2.25$^{h\backslash}$ ± 0.007	0.69^{c} ± 0.01
P1(25 ugmL^{-1})	80.84^{g} ± 1.71	18.56^{f} ± 1.23	8.86^{b} ± 1.11	24.57^{f} ± 0.76	623.9^{f} ± 2.71	43.84^{e} ± 0.86	1.71^{e} ± 0.007	0.732^{e} ± 0.002
P1(50 ugmL^{-1})	73.67^{e} ± 1.84	21.91^{g} ± 0.28	9.45^{c} ± 1.06	25.56^{g} ± 1.11	680.7^{g} ± 3.84	47.17^{g} ± 1.11	2.04^{f} ± 0.008	0.72$^{d\backslash}$ ± 0.001
P1(100 ugmL^{-1})	76.21^{f} ± 1.23	23.08^{h} ± 1.56	13.11^{f} ± 0.54	26.52^{h} ± 0.61	753.1^{h} ± 2.23	45.89^{f} ± 0.61	2.08^{g} ± 0.003	0.74^{de} ± 0.002
